# Incidence and risk factors of cognitive disorders after urologic endoscopic surgery

**DOI:** 10.1192/j.eurpsy.2022.2252

**Published:** 2022-09-01

**Authors:** M. Dahmen, R. Chrigui, A. Loghmari, M. Soussi, S. Mansour, K. Bouassida, M. Kahloul, W. Naija

**Affiliations:** 1 Sahloul Academic Hospital, University of medicine, “Ibn Al Jazzar”, Sousse, Tunisia, Department Of Anesthesia And Intensive Care,, Sousse, Tunisia; 2 University hospital center of Sahloul, Urology Department, Sousse, Tunisia

**Keywords:** -Endoscopique resection, MoCA, Cognitive disorders, Postoperative period

## Abstract

**Introduction:**

Postoperative cognitive disorders are an emerging public health problem because of its related socio-economic impact.: Postoperative cognitive disorders are an emerging public health problem because of its related socio-economic impact.

**Objectives:**

To determine the incidence and risk factors of cognitive disorders after endoscopic resection in urology.

**Methods:**

This is an observational, descriptive and analytical study carried out in the urology department of Sahloul University Hospital during a 3 month period, and enrolling patients scheduled for endoscopic resections. Collected data included socio-demographic characteristics and parameters related to the operative management. Cognitive disorders were assessed by the MOCA Test one day before the intervention, then, during the first postoperative day. Patients developing TURP syndrome were excluded.

**Results:**

During the study period, 104 patients were enrolled with a mean age of 67.76 years. The sex ratio was 33.6. Main interventions were transurethral resection of bladder tumor and transurethral resection of the prostate.The incidence of cognitive disorders was 45.2% after endoscopic resection. Main Risk factors in multivariate analysis were age (p <10-3), low educational level (p <0.001), sedentary (p <0.001), smoking (p = 0.029), an age gap with spouse> 10 years (p <0.001), high blood pressure (p <0.001), myocardial infarction (p = 0.005); chronic bronchitis (p = 0.002), sleep disorders (p <0.001), preoperative concentration disturbances (p = 0.005), poor quality of patient information (p <0.05), and the type of anesthesia (p = 0.012).

**Conclusions:**

The incidence of cognitive disorders after urologic endoscopic surgery is considerable. Patients with risk factors require preventive measures, regular screening and optimal management.

**Disclosure:**

No significant relationships.

